# Speed, slope, and synchrony: Empirical insights into SAR searcher behavior

**DOI:** 10.1371/journal.pone.0339541

**Published:** 2026-06-15

**Authors:** Amanda Hashimoto, Eighdi Aung, Robert Koester, Nicole Abaid

**Affiliations:** 1 Engineering Mechanics Program, Virginia Polytechnic Institute and State University, Blacksburg, Virginia, United States of America; 2 dbS Productions LLC, Charlottesville, Virginia, United States of America; 3 School of the Environment, Geography and Geosciences, University of Portsmouth, Portsmouth, United Kingdom; 4 Department of Mathematics, Virginia Polytechnic Institute and State University, Blacksburg, Virginia, United States of America; 5 Center for the Mathematics of Biosystems, Virginia Polytechnic Institute and State University, Blacksburg, Virginia, United States of America; PLoS ONE, UNITED STATES OF AMERICA

## Abstract

Wilderness search and rescue (SAR) missions are time-critical and terrain-dependent, so planners must quickly allocate resources across complex landscapes. In practice, they rely on expert judgment and experience-based assumptions to coordinate individuals and teams, yet few of these assumptions have been formally validated with field data and modeling. We address this gap by analyzing GPS tracks from 64 SAR incidents, selecting 61 tracks from 13 cases. The tracks are categorized by search tactic: hasty, sweep, or team sweep, with the last divided into six teams of varying size. To quantify how tactic and terrain shape movement, we bootstrapped exponential speed-slope fits, ran Kolmogorov-Smirnov tests, and used a nested ANOVA with random effects. Median uphill and downhill speeds are statistically indistinguishable (0.48 m/s vs. 0.52 m/s; KS *p* = 0.093), suggesting that slope penalties on pedestrian speed can be modeled symmetrically. Hasty searches are faster than sweeps (0.53 m/s vs. 0.39 m/s; KS *p* < 10^−3^), with no interaction between slope and tactic. Team-level analyses using Spearman correlation, time-lagged cross-correlation, and transfer entropy revealed tightly coupled movement and identifiable leaders, with follower reaction lags of only a few seconds. These empirically derived parameters–search-type specific baseline speeds, a single slope coefficient, and realistic coordination bounds–offer practical inputs for SAR coverage calculations and agent-based models. Incorporating values drawn from real field data could refine human mobility assumptions while remaining compatible with the existing SAR operational framework.

## Introduction

Search and Rescue (SAR) outcomes hinge not only on how well we can predict the whereabouts of a lost subject, but also on how efficiently human searchers are able to move, coordinate, and detect clues once on the ground. Time-critical metrics of success such as probability of detection, area coverage, and responder safety all depend on realistic assumptions regarding searcher behavior. These assumptions are often oversimplified or untested in current operation procedures and modeling frameworks [1–4]. This paper attempts to fill that gap by empirically characterizing ground searcher movement and coordination using real-world GPS data.

To quantify the outcome of a search, classical search theory developed three probability metrics; however, these frameworks sometimes lack empirical justification. Originally designed for naval and aerial searches, the approach starts with three key quantities: the *probability of area* (POA), the *probability of detection* (POD), and the *probability of success* (POS). POA is the prior probability that the subject is actually in the piece of terrain being searched, while POD is the conditional probability the searchers would detect the subject *if* the subject is there. POD, in turn, depends on how thoroughly the area is covered (often summarized by a coverage value *C*) and on how detectable the subject is. POS is the overall chance the search will find the subject and is thus defined as POA×POD=POS. Effective sweep width (*W*) captures detectability: it is the width of an idealized strip that would yield the same expected detections as a real, imperfect searcher. Lateral range curves describe how detection probability falls off with distance from the search path and are one way to estimate *W*. The WWII-era kinematic and detection models of Koopman and later operations-research work by Charnes and Cooper formalized these relationships and how to allocate effort across areas [5–7]. In land SAR practice, however, these frameworks are adapted with correction factors and rule-of-thumb spacing tables, while key variables such as speed are typically treated as inputs or constants rather than measured outcomes [3,4,8–10]. Recent field studies show that simple spacing heuristics (e.g., “head, belt, boots”) can yield consistent POD across different environments [11], but these advances still assume nominal searcher speeds rather than empirically derived, tactic- and terrain-specific values.

SAR training manuals codify tactics like hasty, route, and sweep searches, but offer only heuristic guidance on expected speeds or team spacing, and rarely ground those guidelines in large GPS datasets [12,13]. Even simulation studies of searcher performance often assume perfect detection or distance-only effects [14]. As a result, planners may rely on unvalidated parameters for mobility and simplified detection models.

In contrast to this lack of empirical work on human searchers, there is a rich literature on lost person behavior (LPB). Researchers have proposed statistical priors, agent-based models, and space-time risk maps incorporating environmental context, subject goals, and evidence cues [15–21]. Yet in many of these same frameworks and in subsequent simulations, searchers are reduced to homogeneous, constant-rate coverage agents, ignoring the human variability and team dynamics that influence real-world operations [22,23].

Outside of the context of SAR, decades of work have examined how slope, terrain, and physiology shape human locomotion. From Naismith’s rule and Tobler’s hiking function to large-scale GPS analyses and laboratory energetics studies, robust evidence shows that walking and running speeds vary systematically with grade and substrate [24–29]. However, these studies are rooted in recreational or controlled contexts; they do not differentiate between tactical search modes, nor do they examine coordination within small teams performing cognitively demanding tasks. A search team has to be able to move through the environment, often moves off-trail, needs to navigate, and, perhaps most importantly, must move at a speed that allows searching for small clues in all directions. Related efforts in outdoor mobility or recreation mapping highlight spatial and temporal use patterns but not SAR-specific tactics [30].

SAR-specific mobility modeling has progressed through GIS-based least-cost paths, travel-time cost-surface models, and integrated toolkits such as the National Park Service Travel Time Cost Surface Model (TTCSM) and *movecost* R software package [31–37]. These techniques demonstrate how terrain, landcover, and infrastructure shape predictions in movement, yet few have been validated against actual team GPS tracks during missions. Fewer still distinguish movement parameters by search tactics (e.g., hasty vs. sweep), account for group coordination, or incorporate empirically measured reaction lags and information flow.

Meanwhile, studies of collective motion, leadership, and coordination in pedestrian and human-robot teams provide quantitative tools, like cross-correlation, transfer entropy, and anticipatory planning models, for detecting leader-follower dynamics and synchronous behavior [38–46]. Studies involving community walking groups also suggest that leaders may differ in psychological traits rather than just activity levels [47]. Still, these methods have rarely been applied to ground SAR teams operating in rugged environments. This leaves open questions about how tightly searchers coordinate, how quickly they react to one another, and whether identifiable leaders drive team movement patterns.

This study therefore fills several gaps. First, it provides a side-by-side empirical comparison of hasty and sweep tactics using real GPS tracks. Second, it quantifies uphill versus downhill effects on searcher speed and tests for symmetry within those effects. Third, it documents intra-team coordination within small search groups, specifically speed coupling, spatial proximity, reaction lags, and directional information flow. Fourth, these observed patterns can be translated into parameters to be used in SAR mobility, coverage, and agent-based models [48–51]. We will return to these modeling implications in the discussion. Accordingly, we ask the following Research Questions:

1. How does elevation (uphill vs. downhill) affect searcher speeds?
2. How do different search types (sweep vs. hasty) influence searcher speed and movement patterns?
3. Do grouped searchers exhibit correlated behavior and identifiable leader-follower dynamics?

The remainder of this paper is organized as follows: The Materials and methods section first describes the dataset and pre-processing (alignment, interpolation, spike handling), then details the statistical and information-theoretic analyses (bootstrapping, exponential fits, ANOVA, cross-correlation, transfer entropy). The Results section presents findings for elevation effects, search tactic comparisons, and team coordination. The Discussion section examines implications for SAR planning and modeling, limitations, and future directions. Lastly, the Conclusions section discusses applications and recommendations for integrating these findings into operational tools.

## Materials and methods

### Search incident data

This study utilizes a dataset of 64 SAR incidents that occurred between 2020 and 2023 in Virginia, USA. The incidents span a range of geographic settings, including rural and wilderness areas characterized by hilly, mountainous, and flat terrains. Each incident is geo-referenced with latitude and longitude coordinates detailing various components of the search planning process, specifically planned search regions, Initial Planning Points (IPPs), clues, and GPS tracks of the individual searchers and search teams. Each search track includes coordinates, timestamps, and elevation data. From this extensive dataset of SAR incidents, we selected 13 distinct cases for this study. These 13 incidents comprise 61 different search tracks, which we have organized into three primary categories: hasty, sweep, and team sweep. Hasty searches are characterized by tracks where the searcher typically traverses a linear feature, whereas sweep searches are distinguished by a grid-like, back-and-forth movement across an area. Tracks that are visually identified as moving in parallel paths within the same search area and overlapping in time (typically within a temporal window of a few minutes and spatial separation of 20–40 meters) are classified as paired or team sweep searches.

**Fig 1:**
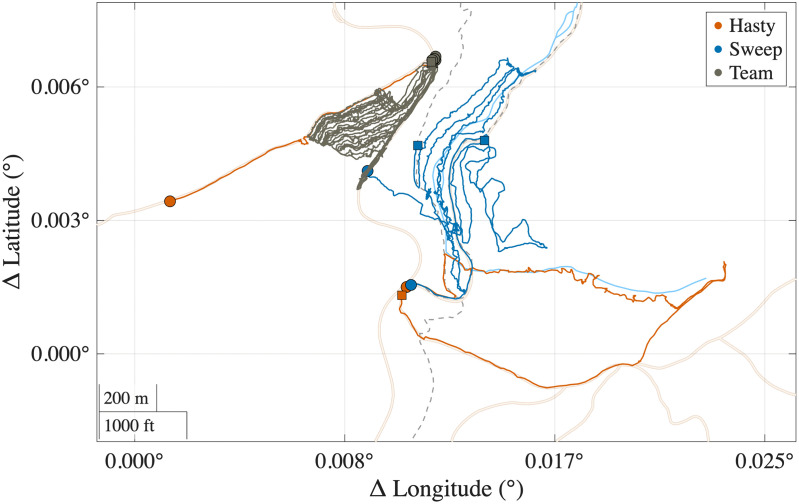
An example of a search incident. The hasty (orange), sweep (blue), and team (gray) searches are shown on the map, where start and end positions are in corresponding colored circles and squares, respectively. Search trajectories are overlaid on transportation and hydrography layers derived from publicly available U**.**S. Geological Survey (USGS) National Map data.

Due to the diverse sources of incidents and GPS trackers employed, the dataset exhibits substantial noise. To curate our selection of 61 tracks, exclusion criteria are applied to eliminate cases that did not resemble simple human walking movement (for example, they may have been collected from searchers using ATVs or aerial drones). By utilizing the latitude and longitude coordinates of each timepoint, we calculated the distance, time, and elevation differences between steps. Each successive step will be referred to as a track segment. Segment speeds are derived as the quotient of distance over time, while slope was determined as the elevation difference over the distance. Consequently, tracks exhibiting a mean speed exceeding 2.5 m/s or a maximum speed surpassing 4 m/s, indicative of vehicular movement, are excluded. Additionally, tracks with over 100 timesteps showing no movement are omitted. Despite these exclusion criteria, instances persisted where spikes in speed or slope values were observed. Tracks with less than 1% of segments identified as spikes were retained and subjected to a cleaning procedure. Instances where the speed exceeded 5 m/s or the 99.9^th^ percentile of that track’s speed distribution were labeled as spikes. Similarly, instances where the absolute value of the slope surpassed 5 or the 99.9^th^ percentile of the corresponding track’s slope distribution were labeled as spikes. To rectify these spikes, elevation, distance, and time values are interpolated linearly between timepoints immediately preceding and following the spike, followed by recalculating speeds and slopes. The refined tracks comprised 20 hasty, 23 sweep, and 18 sweep team tracks, where the team tracks are divided into 6 distinct groups. Fig 1 illustrates an incident featuring hasty (orange), sweep (blue), and team (gray) search tracks. Hasty searchers proceed along linear features, such as roads or trails, whereas sweep and team sweep searches navigate back and forth within a designated area.

### Ethics statement

The data analyzed in this study consist of pre-existing GPS tracking data collected during routine search and rescue training and operational activities. The present study involved secondary analysis of these previously collected data. The researchers did not interact with human participants, and the dataset was obtained in de-identified form with no personally identifiable information (PII).

Collection of these operational data occurred under prior work conducted in coordination with the U.S. Department of Homeland Security (DHS) Science and Technology Directorate and did not include PII.

### Statistical metrics

#### Bootstrapped Kolmogorov-Smirnov test.

In order to understand the underlying patterns within the search data, we can use a number of different statistical metrics to assess the influence of landscape and search strategy on speed. Given the inherent variability in the total length of each track, we must utilize methods that account for the variable number of data points per track. This can be achieved through a bootstrapping technique that ensures equal representation of all tracks.

The initial metric integrates bootstrapping with the Kolmogorov-Smirnov (KS) test to differentiate the distributions between uphill and downhill speeds and hasty versus sweep speeds. Bootstrapping involves repeatedly resampling the sample data with replacement to generate a number of bootstrapped samples. By using the minimum number of uphill and downhill segments (or hasty and sweep segments), this number of segments is sampled 1000 times so all tracks are equally represented. The KS test is applied to these equal-weighted samples for each bootstrap iteration, resulting in a median *p*-value that determines the significance between uphill and downhill (or hasty and sweep) speed distributions. Because segment-level observations within a track are likely to be correlated, these KS comparisons are interpreted as distribution-level contrasts rather than as strictly independent segment-level inference. To provide track-level inference that treats each track as the independent unit of analysis, we additionally performed paired Wilcoxon signed-rank tests on per-track median speeds when comparing uphill and downhill conditions.

#### Exponential fitting.

We also leverage bootstrapping to model the uncertainty between tracks and within tracks. To see the differences in the effect of moving on uphill or downhill slopes on searcher speed, we first fit the observed speeds with an exponential curve, defined as


$$\begin{array}{c} v=ae^{bx}, \end{array}$$ (1)

where *v* is the speed, *a* is the intercept parameter that represents the speed at slope zero, *b* is the slope effect parameter (*b* < 0 for uphill and *b* > 0 for downhill in the fitted results), and *x* is the observed slopes at each segment of the track. Exponential fits are performed separately for uphill and downhill subsets without imposing sign constraints on *b*; the sign of the coefficient emerges from the data. To prevent over-representation of longer tracks, a two-level bootstrapping approach is employed to assess the uncertainty in each fitting parameter. During each of the 1000 bootstrap iterations, tracks are randomly selected with replacement, followed by the random selection of segments within each track, also with replacement, and subsequently aggregated for model fitting. This two-level resampling accounts for the hierarchical structure of the data, capturing both inter-track and intra-track variability without assuming independence of segment-level observations across tracks. The set of 1000 bootstrapped parameters is then utilized in Eq 1 to generate 1000 distinct bootstrap curves. The 2.5% and 97.5% percentiles of these curves are subsequently computed to establish the confidence intervals for the initial fits. This approach provides parameter estimates and confidence intervals that fairly capture both the inter- and intra-track variability. The application of this method to both uphill and downhill speeds facilitates direct comparisons to understand how slope effect influences searcher speed.

#### Nested analysis of variance.

While the previous metrics make separate comparisons of elevation and search type with respect to speed, we aim to comprehensively analyze the extent of the slope effect *between* search types. This analysis is facilitated through a Nested Analysis of Variance (ANOVA) incorporating random effects [52]. The mean search speed of each track serves as the dependent variable, whereas the independent variables comprise the search type (hasty or sweep), slope direction (uphill or downhill or flat), and the track itself. The track is treated as nested within the searcher and constitutes a random effect, considering each searcher is distinct and represents a sample from the broader population of potential searchers. This approach enables a three-level categorical comparison to elucidate differences in speed across uphill, downhill, and flat terrains between hasty and sweep searches. The dataset satisfies ANOVA assumptions, including independence, residual normality, homogeneity of variances, and normality of random-effect distributions. To evaluate the pairwise differences between slope categories, we conduct three post hoc two-sample t-tests. However, when making several comparisons, the potential inflation of Type I error due to multiple comparisons is mitigated by applying the Bonferroni correction to the *p*-value, thereby maintaining an overall Type I error rate of 5% [52,53].

#### Spearman correlation.

To investigate coordination within team searches, we perform a Spearman rank correlation (ρ) analysis of teammate speeds within groups. First, we align each searcher’s data to a common time window within each team, linearly interpolate latitude, longitude, and elevation variables to a 1 Hz temporal grid, recalculate differences in distance and elevation based on these interpolated variables, and subsequently recalculate speed and slope using these updated differences. This approach facilitates time-aligned comparison of each team member’s speed. We then employ Spearman rank correlation to test for monotonic associations between team members’ speeds, elucidating any synchronous changes in teammate velocities. To account for temporal autocorrelation, we downsample the speed time series of each team by δ using the decorrelation timescale (1/*e* threshold) we obtain from the average autocorrelation function (see S1 Table for details). Spearman correlations are computed using the downsampled time series.

#### Time-lagged cross-correlation.

Following the assessment of speed synchrony between teammates, we analyze the latency in teammates’ responses to speed changes and evaluate how closely speeds align once synchronized. For each teammate pair, we first remove the mean speed from both tracks to center the time series around zero. Then, using MATLAB’s *xcorr* function [54,55], we compute the cross-correlation of detrended speed series across a time lag interval from −60 seconds to +60 seconds. The maximum correlation is identified at the time lag where the absolute cross-correlation value peaks. This lag indicates how much one individual’s speed variation follows another’s: zero lag suggests simultaneous speed changes, a positive lag indicates the second individual’s speed changes lag behind the first by that number of seconds, while a negative lag indicates the opposite. The peak cross-correlation magnitude represents the strength of the reaction.

This time-lagged cross-correlation analysis can also be applied using cumulative distances traveled to determine leadership dynamics within groups. Rather than comparing speeds, we calculate cumulative distances for each track by integrating step-lengths between successive 1-second intervals, constructing monotonic distance curves. For each pair of teammates, we again detrend and then perform cross-correlation on the vectors over the same time lag of ±60 seconds. We find the maximum correlation and the sign of the peak lag indicates who typically moves ahead first. In order to elect the leader of each team, votes are assigned to the individual consistently moving ahead with a lag greater than one second. When lag differences are one second or less, indicating close synchronization, we utilize a tie-breaking *percent-ahead* metric defined as


$$\begin{array}{c} P_{A<B}=\frac{time(d_{A}(t)>d_{B}(t))}{total\;time}, \end{array}$$ (2)

where *d*_*A*_(*t*) and *d*_*B*_(*t*) represent the cumulative distance vectors for teammates *A* and *B*, respectively. The teammate who remains ahead at least 51% of the time receives the vote. The individual with the majority of votes from pairwise comparisons is designated as the team’s leader.

#### Transfer entropy.

To quantify directional coupling between a team’s elected leader and each follower, we can use transfer entropy (TE), an information-theoretic measure introduced by Schreiber in 2000 [56]. Building on Shannon’s concept of entropy, which captures the uncertainty in a time series, TE quantifies how much uncertainty is reduced in predicting a time series from its own past history by also knowing the past history of another time series. Formally, for two discrete Markov processes *X* and *Y*, the transfer entropy from the source time series *X* to the target time series *Y* with lag τ is given by


$$\begin{array}{c} {TE}_{X\to Y}=\sum p(y_{t},\;y_{t-1},\;x_{t-\tau })\log\frac{p(y_{t}|y_{t-1},\;x_{t-\tau })}{p(y_{t}|y_{t-1})}. \end{array}$$ (3)

In our analysis, we quantify the influence of the leader’s speed, represented by *X*, on each follower, represented by *Y*, by computing transfer entropy. The leader’s speed serves as the source, and any follower’s speed serves as the target time series. To satisfy the Markov property of the time sequences, we downsample the data by computing the mean speed at one-second intervals. We utilize the JIDT toolbox [57] to compute transfer entropy, with the time delays (τ) set according to results from the cross-correlation analysis. The JIDT toolbox has an option to apply the Kraskov, Stögbauer, and Grassberger (KSG) [58] algorithm, which estimates the probabilities based on binning *k* nearest neighbors, where we fix *k* = 2. Since the magnitude of $TE_{X\to Y}$ alone is arbitrary, we establish a statistical control for comparison. A standard approach [59,60] involves randomly shuffling the source time series (*X*), effectively eliminating its temporal structure and consequently its influence on the target (*Y*). To preserve some local structure of the source time series, we generate 1000 random shuffles of *X* by randomly permuting the source time series in blocks of 60 seconds. Each shuffled replicate provides an associated TE value relative to *Y*. It is possible to obtain negative values for transfer entropy, which indicates that the relationship is less than the expected value if the variables were not related [61]. Consequently, negative TE values are considered to be zero.

We evaluate the statistical significance of the observed TE against the distribution of the shuffled TE values ($\mu_{control}$) by using a *z*-test. By integrating TE with cross-correlation and reaction lag analyses, we can not only identify who walks in front or who accelerates first, but also determine whose speed history actively drives subsequent follower movements.

## Results

### Effects of slope on speed

**Fig 2:**
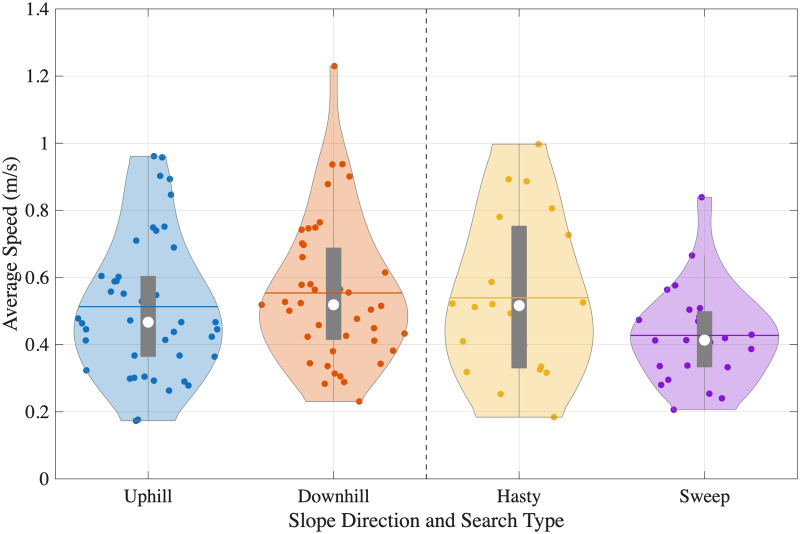
Uphill, downhill, hasty, and sweep speeds. For uphill, downhill, hasty, and sweep categories, the median speeds (white circles) shown with their respective IQRs (gray boxes), means (horizontal colored line), and spread.

To address Research Question 1, we quantified the influence of elevation on speed by separating all track segments into uphill (slope < 0) and downhill (slope > 0) segments. Due to varying track lengths, we utilized medians rather than means to compute representative speeds. Median uphill speed is 0.48 m/s (interquartile range [IQR], the range between the 25^th^ and 75^th^ percentiles, is 0.26 − 0.77), and median downhill speed is 0.52 m/s (IQR 0.30 − 0.85), which are displayed as violin plots on the left side of Fig 2. To evaluate if this difference is statistically significant, we compared the per-track median speed distributions using a bootstrapped KS test, ensuring each track has an equal weight. With a minimum of 10 segments per track for both uphill and downhill conditions, our analysis revealed no statistically significant difference between uphill and downhill speeds (1000 bootstraps; median KS *p* = 0.093, with 38.5% of bootstrap iterations yielding *p* < 0.05). A paired Wilcoxon signed-rank test on per-track median speeds similarly indicated no statistically significant difference between uphill and downhill conditions (*p* = 0.078).

**Fig 3:**
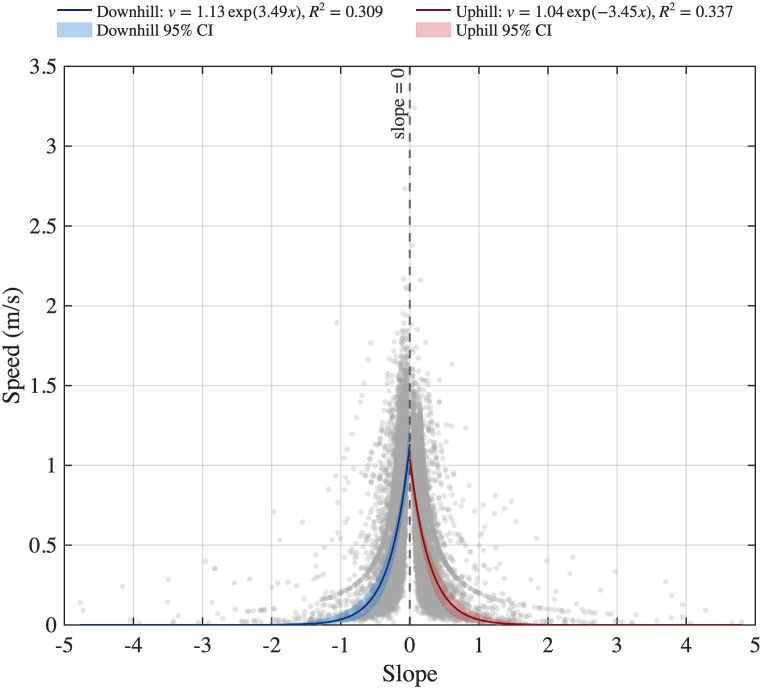
Uphill and downhill exponential fits. The exponential fit curves for all uphill and downhill segments shown with 95% confidence intervals for downhill (blue) and uphill (red) bootstrapped parameters.

Additionally, since searcher speed exhibits exponential decay or growth relative to slope, we examined differences in the magnitude of this relationship. Performing a two-level bootstrap analysis, we fitted Eq 1 separately to uphill and downhill speeds. Our findings indicated similar coefficients for both conditions. The uphill speed fit using per-track medians yielded v=1.04exp(−3.45x) (*R*^2^ = 0.337), while the downhill fit yielded v=1.13exp(3.49x) (*R*^2^ = 0.309). The flat-ground speed parameter *a*, which reflects the modeled speed at zero slope, had a mean value of 1.046 (95% CI 0.901 − 1.190) for uphill and 1.130 (95% CI 0.944 − 1.298) for downhill conditions. Although this parameter corresponds to slope = 0, its value can differ between conditions because each exponential fit was fit only to positive or negative slope data, thus it reflects extrapolation from those fits rather than observed measurements on flat ground. The slope effect parameter *b* had a mean of −3.503 (95% CI −4.735 to −2.619) for uphill and 3.488 (95% CI 2.642 − 4.624) for downhill. The fitted exponential curves, along with bootstrap-derived confidence intervals, are shown in Fig 3, displaying uphill (right, red interval) and downhill (left, blue interval) fits. Table 1 summarizes these parameter values for all tracks in the first row. The overlapping confidence intervals between uphill and downhill conditions suggest no statistically significant differences in the effect of uphill or downhill slopes on speed.

**Table 1:** Exponential speed-slope fits ($v=ae^{bx}$) and bootstrapped parameter means (95% CI).

				Mean of bootstrapped parameters			
Type	Slope	Exponential fit	*R* ^2^	*a*	95% CI	*b*	95% CI
All	Uphill	*v* = 1.04 *e*^–3.45*x*^	0.337	1.05	[0.90, 1.19]	−3.50	[−4.74, −2.62]
	Downhill	*v* = 1.13 *e* ^3.49*x*^	0.309	1.13	[0.94, 1.30]	3.49	[2.64, 4.62]
Hasty	Uphill	*v* = 1.11 *e*^–3.18*x*^	0.347	1.11	[0.92, 1.27]	−3.21	[−4.49, −1.93]
	Downhill	*v* = 1.20 *e* ^2.96*x*^	0.287	1.17	[0.93, 1.39]	2.94	[1.73, 4.16]
Sweep	Uphill	*v* = 0.88 *e*^–3.21*x*^	0.307	0.93	[0.80, 1.14]	−3.57	[−5.61, −1.98]
	Downhill	*v* = 0.94 *e* ^3.21*x*^	0.294	0.97	[0.84, 1.16]	3.48	[2.12, 5.28]

**Fig 4:**
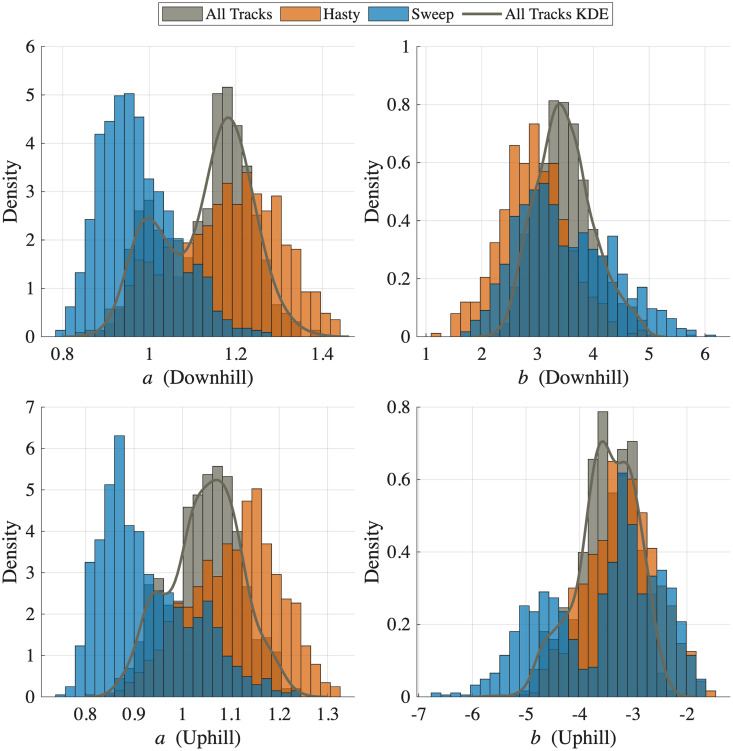
Bootstrapped parameter distributions. The exponential fit bootstrapped parameter distributions *a* and *b* for hasty (orange), sweep (blue), and all tracks (gray) for both the uphill (bottom row) and downhill (top row) fits.

It should be noted that this fitting included all segments from both uphill and downhill categories. In the process of bootstrapping the parameters, we observed bimodality in the distribution of the flat-ground speed parameter *a*. Further investigation revealed differences in the parameter distributions between hasty and sweep search types, necessitating separate consideration of uphill and downhill speeds by search type in subsequent analyses. Fig 4 displays the parameter distributions for hasty, sweep, and all combined tracks, separated by uphill (bottom row) and downhill (top row) segments for parameters *a* and *b*. Specifically, in the left column for parameter *a*, the hasty (orange) and sweep (blue) were slightly skewed left and right, respectively, resulting in the bimodal distribution observed in the all track histogram (gray).

### Search type comparison

As demonstrated in the previous section, search type appears to influence searcher speeds differently. To address Research Question 2, we initially separated tracks into hasty and sweep search categories to calculate overall median speeds. Median speeds were 0.53 m/s (IQR 0.26 − 0.87) for hasty searches and 0.39 m/s (IQR 0.20 − 0.63) for sweep searches, illustrated on the right-hand side of Fig 2. To evaluate the statistical significance of these differences, we performed the bootstrapped KS test to account for variations in track lengths. Utilizing a minimum of 112 segments per track for both hasty and sweep searches, we identified a statistically significant difference between the two search types (1000 bootstraps; median KS *p* < 10^−3^, with 100% of bootstrap iterations yielding *p* < 0.05). However, as this test was performed on aggregated median speeds without separating uphill and downhill segments, caution should be exercised when interpreting this result.

**Fig 5:**
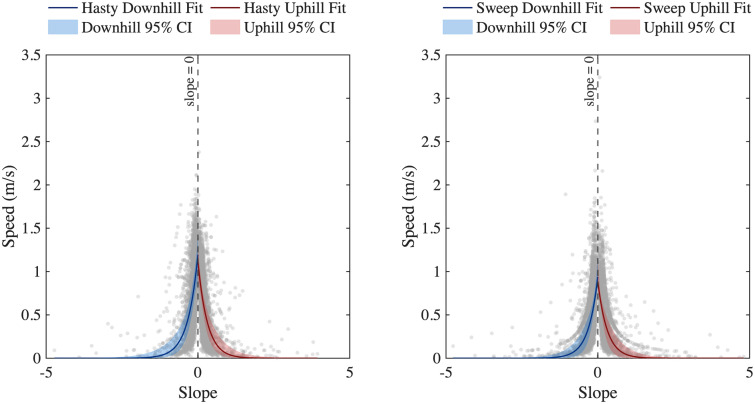
Hasty and sweep exponential fits. The exponential fit curves for hasty (left) and sweep (right) uphill and downhill segments shown with 95% confidence intervals for downhill (blue) and uphill (red) bootstrapped parameters.

Given the bimodality observed in the elevation-dependent parameter distributions (see Fig 4), we further separated each search type into uphill and downhill slope segments. Employing a two-level bootstrap analysis, we fit the exponential Eq 1 to uphill and downhill segments for both search types. For hasty searches, the uphill speed fit was v=1.11exp(−3.18x) (*R*^2^ = 0.347) and the downhill speed fit was v=1.20exp(2.96x) (*R*^2^ = 0.287). The flat-ground speed parameter *a* had a mean of 1.107 (95% CI 0.920 − 1.267) for uphill segments and 1.173 (95% CI 0.927 − 1.389) for downhill segments. The slope effect parameter *b* exhibited a mean of −3.213 (95% CI −4.494 to −1.928) for uphill and 2.935 (95% CI 1.729 − 4.163) for downhill segments. The exponential fit curves and confidence intervals for uphill and downhill hasty tracks are shown on the left side of Fig 5. For sweep searches, the uphill speed fit was v=0.88exp(−3.21x) (*R*^2^ = 0.307), and the downhill speed fit was v=0.94exp(3.21x) (*R*^2^ = 0.294). The flat-ground speed parameter *a* had a mean of 0.926 (95% CI 0.795 − 1.136) for uphill and 0.973 (95% CI 0.838 − 1.158) for downhill. The slope effect parameter *b* had a mean of −3.573 (95% CI −5.614 to −1.983) for uphill and 3.480 (95% CI 2.115 − 5.281) for downhill segments. The right side of Fig 5 shows the exponential fits for sweep search tracks. The equations and parameter values for both search types are summarized in Table 1, with hasty tracks in the second row and sweep tracks in the third row. In both search categories, overlapping of the absolute values of the confidence intervals between uphill and downhill conditions indicate no statistically significant difference in the effect of slopes on searcher speed.

### Search type and slope effects on speed

Having individually explored the effects of elevation and search type on searcher speed, we now evaluate the combined effect of search type and slope direction on speed. This analysis is conducted utilizing a nested ANOVA incorporating random effects, where the track acts as the random effect nested within the search type. The two categorical fixed effects considered are search type (hasty or sweep) and slope direction (flat, uphill, or downhill).

**Fig 6:**
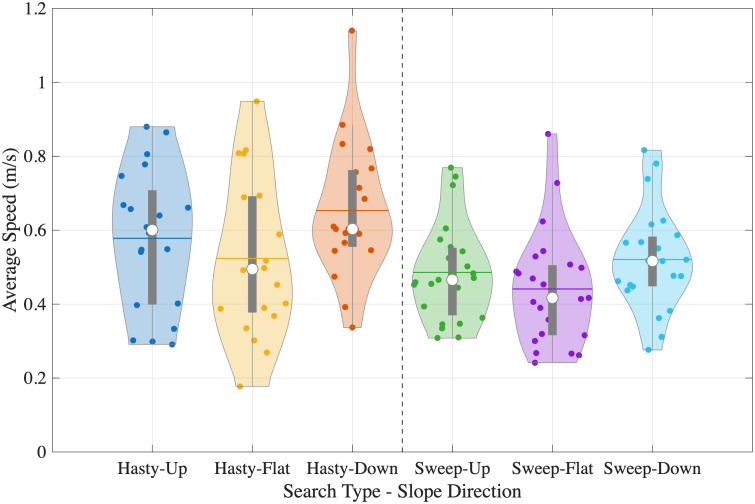
Uphill, downhill, and flat speeds by search type. For search types, hasty (left) and sweep (right), violin plots indicate the median speeds (white circles) shown with their respective IQRs (gray boxes), means (horizontal colored line), and spread for uphill, downhill, and flat segments, respectively.

Our analysis indicates a significant difference in overall speeds between hasty and sweep searches (*F*(1,41) = 4.85, *p* = 0.03), with mean speed strongly dependent on slope direction ($F(2,82)=15.51,\;p<10^{-3}$). The interaction between slope direction and search type was not significant (*F*(2,82) = 0.98, *p* = 0.38), suggesting consistent effects of slope direction across both search types. Additionally, substantial variability was observed in mean speed between individual searchers ($F(41,82)=9.16,\;p<10^{-3}$). Fig 6 illustrates these findings with violin plots depicting the speed distributions across all combinations of search type and slope direction.

To further evaluate the differences between slope categories, we performed three two-sample t-tests, applying a Bonferroni correction to the resulting *p*-values. Speeds during downhill segments were on average 0.103 m/s (95% CI 0.015 − 0.191) faster compared to flat segments, a statistically significant difference (*p* = 0.0167). Although downhill speeds were also faster on average by 0.054 m/s (95% CI −0.034−0.142) compared to uphill segments, this difference was not statistically significant (*p* = 0.3258). Speeds on flat segments were, on average, slower by 0.049 m/s (95% CI −0.137−0.039) compared to uphill speeds; however, this difference also did not reach significance (*p* = 0.3868). These results suggest that teams, irrespective of employing hasty or sweep strategies, tend to slow down on flat and uphill segments compared to downhill segments. Additionally, hasty searches consistently demonstrated higher average speeds compared to sweep searches.

### Group search team behaviors

**Fig 7:**
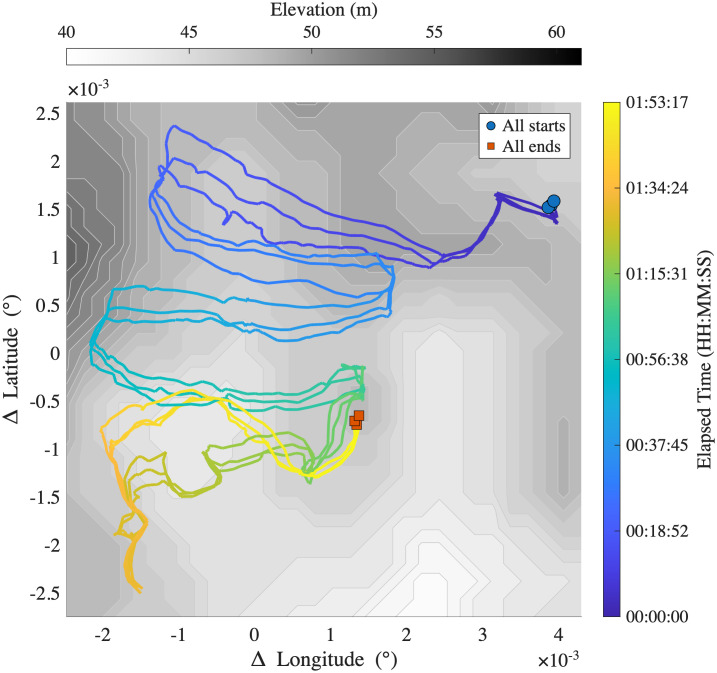
Team 1 search track coordinates. The search tracks for Team 1 shown over their common interpolated time from beginning (blue) to end (yellow), indicated by the right colorbar. The markers show the track starts (blue circles) and ends (red squares). The background shows the elevation of the region using the top colorbar to show the elevation range, where darker shades of gray represent higher elevations with respect to sea level.

Using team tracks, we address Research Question 3 to uncover underlying coordination among teammates. Specifically, we seek to identify patterns in spatial proximity, investigate correlations in speed and distance, and discover potential leader-follower dynamics. Our analysis includes 18 searcher sweep tracks organized into 6 distinct teams. After aligning tracks to a standardized time frame and recomputing speed and slope at each timestep for direct comparison, we conducted a Spearman rank correlation analysis on speeds between each pair of searchers within the teams. To account for temporal autocorrelation in the 1 Hz time series, we estimated decorrelation timescales for each team and recomputed Spearman correlations using downsampled series separated by these timescales. The resulting median correlation (ρ=0.78, IQR 0.71 − 0.85 across teams) indicates that half of the searcher pairs exhibit a strong coupling in acceleration, wherein acceleration by one teammate typically elicits similar acceleration from another. Time-lagged cross-correlation of teammate speeds revealed a median optimal lag of 2 seconds (IQR −2−6), with approximately 90% of reaction lags less than 10 seconds. Furthermore, the strength of these correlations at the identified optimal lags demonstrated strong coupling (median |*r*| = 0.64, IQR 0.55 − 0.71). The median mean spatial separation between teammates was 17 meters (IQR 15−26), indicating that teams will often maintain close visual contact. Although pairs occasionally exhibited larger separations (median maximum separation of 71 meters, IQR 51 − 94), these gaps were typically brief, given the relatively small mean separation. Fig 7 illustrates the temporal and spatial progression of one team’s search pattern, highlighting synchronized, sweep-like movement. Track visualizations for Teams 2–6 are shown in S1–S5 Figs.

To identify leadership dynamics within teams, we analyzed cumulative distances traveled by each teammate, through pairwise time-lagged cross-correlation, assessing which teammate consistently advanced first. The individual receiving the most votes, determined by the frequency of their reaction lag exceeding that of other teammates, was designated as the leader. In the event of a tie, the teammate physically positioned ahead (highest cumulative path-distance) for over 51% of the track length was awarded the vote. Using this criterion, we successfully identified leaders in all six teams. A summary of all leader-follower pair metrics can be found in S1 Table.

**Fig 8:**
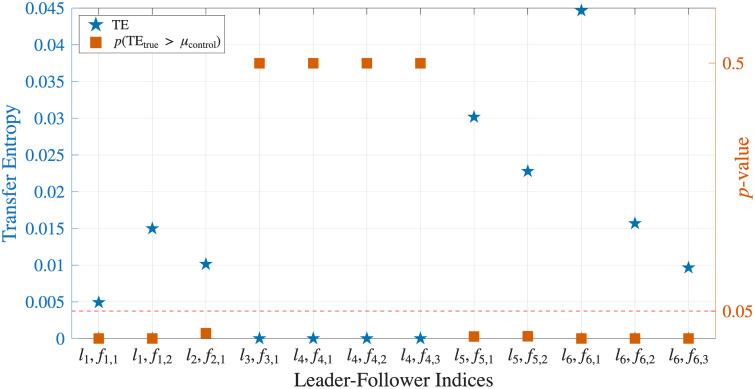
Transfer entropy and *p*-values for every leader-follower pair. Each leader-follower pair is denoted by *l*_*i*_, *f*_*i*,*j*_ where teams are denoted by i=1,…,6 and *j* represents their respective followers. The transfer entropy values (blue stars) for each of the leader-follower pairs and their respective *p*-values (orange squares) for when the observed TE values exceed the mean of the randomized control.

**Fig 9:**
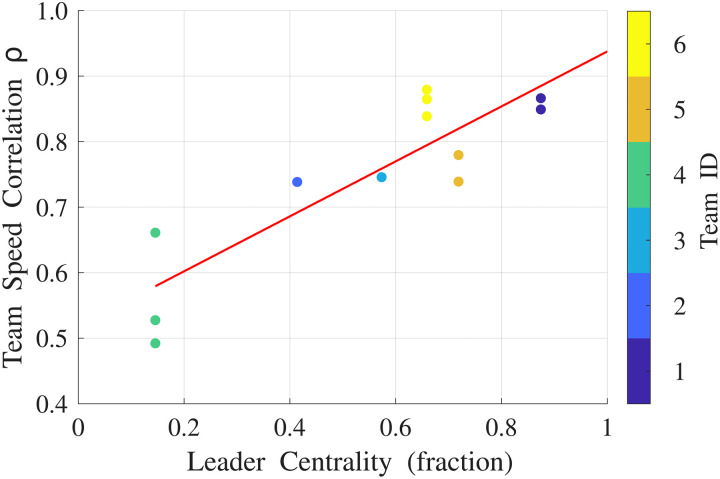
Team speed correlation and leader centrality. For each of the 6 teams, we have the Spearman speed correlation ρ versus the fraction of the track time that the leader was near the center of the group. The red line shows the correlation between the variables with *R*^2^ = 0.77.

We validated the positional interactions between identified leaders and followers through transfer entropy analysis. Fig 8 presents TE values and associated *p*-values for each leader-follower pair, assessing whether the observed TE values exceeded the mean of the shuffled controls. The leader-follower pairs are denoted as *l*_*i*_, *f*_*i*,*j*_, with i=1,…,6 representing each team and *j* representing followers within each team. Positive TE values were identified in Teams 1, 2, 5, and 6, indicating directional information flow between leaders and followers. Conversely, Teams 3 and 4 exhibited negligible TE values, suggesting there was less directional information exchange. These findings correlate with additional metrics such as team speed correlation ρ and leader centrality. Specifically, Fig 9 indicates that Team 4’s leader maintained central group positioning only 14% of the time, coinciding with the lowest observed speed correlation, and thereby corroborating the minimal TE observed for this team.

## Discussion

This study examined multiple variables influencing searcher speed and group behaviors during SAR operations. Because classical planning metrics (e.g., POA×POD=POS) ultimately depend on how thoroughly and quickly teams cover terrain, these empirical patterns matter not just descriptively but for how we estimate coverage (*C*), POD, and thus overall success. Our investigation is structured into four key parts: effects of elevation on speed, differences between search types, interactions between search type and slope, and dynamics within group searches. Below we discuss each in detail and interpret the implications.

### Uphill and downhill slopes affect search speed symmetrically

When we considered per-track median speeds and bootstrapped segment-level comparisons, our analysis revealed no statistically significant differences between uphill and downhill searcher speeds. Although the raw median speeds were slightly higher downhill (0.52 m/s) compared to uphill (0.48 m/s), this discrepancy was not statistically significant, consistent with the relatively symmetrical exponential fits. The uphill and downhill exponential coefficients exhibited very similar magnitudes (both |b|≈3.5), suggesting that the slope direction may have a balanced effect on searcher speed. This symmetry in slope simplifies the incorporation of elevation effects in predictive modeling of search behavior, because it allows for a single magnitude parameter to describe both uphill and downhill movements.

However, we also identified a bimodal distribution in the flat-ground speed parameter *a*, which prompted further investigation that revealed some notable differences between hasty and sweep search types. There is also some indication of a second bimodal distribution in the uphill slope coefficient *b*, as illustrated in Fig 4 in the bottom-right panel. This seems to occur only in the sweep search type, suggesting that sweep searches have distinct behavioral attributes. The contrast could be due to a number of factors, from task variation on different terrain types, such as open flat areas versus steeper hills, to the literal biomechanical differences among searchers which may cause them to move with one of two distinct behavior types. Therefore, while elevation effects alone appear to be symmetrical and consistent across slopes, the interaction with search strategy could complicate modeling and should be explicitly addressed.

We should also note that zero-slope track segments were excluded from the exponential fitting procedure when estimating the flat-ground speed parameter *a*. Instead, the intercept was derived by extrapolation from the fitted model. These segments usually exhibited disproportionately low speeds, reflecting stationary or minimal movement rather than normal flat-ground walking speeds, likely associated with pauses for searching, consultation, or GPS measurement noise. Therefore, in order to avoid biasing towards very low speed estimates, flat segments were omitted from the fitting, with the modeled intercept at zero slope as the best estimate of typical flat-ground walking speeds.

### Hasty searches are faster than sweep searches

In analyzing the search types separately, we found that hasty searches exhibited significantly faster overall speeds compared to sweep searches, with median speeds of 0.53 m/s versus 0.39 m/s, respectively. This difference was statistically confirmed by the bootstrapped KS test. This implication that strategy impacts movement speed also aligns with operational SAR missions, wherein hasty searches are expected to cover ground more rapidly to quickly identify clues, whereas sweep searches adopt a more methodical and thorough approach to ensure full coverage of an area [13]. Additionally, although hasty searches generally demonstrated higher overall speeds, their wider interquartile range, as seen in Fig 2, suggests a greater variability compared to sweep searches.

Moreover, the exponential fitting of parameters revealed differences in the flat-ground speed parameter *a* between search types. Specifically, hasty searchers consistently displayed higher flat-ground speeds than sweep searchers, which explains the bimodality we observed in the parameter distribution when combining all tracks. Regardless of this difference in baseline speeds, the slope effect parameter *b* was similar between both search types, which suggests that search strategy mainly affects the baseline speed rather than sensitivity to slope changes. This interpretation is further supported by the statistically insignificant differences between uphill and downhill slope effects on search speed within both hasty and sweep types, aligning with our earlier results of symmetrical slope impacts.

### Slope influences speed consistently across search types

After examining the individual effects of search type and terrain on speed, we quantified their combined influence using a nested ANOVA approach. This analysis revealed significant differences in search speeds related to search type and slope direction. Notably, searchers moved significantly faster downhill than on flat ground, whereas differences in downhill versus uphill and uphill versus flat speeds were not statistically significant. This result suggests that slope only has a moderate effect on searcher speeds. More importantly, our analysis confirmed no significant interaction between the slope direction and search type, thereby reinforcing the conclusion that slope influences speed consistently regardless of the employed search strategy.

### Search teams show strong coordination and spatial proximity

Our investigation of group searches aimed to understand coordination patterns, leadership dynamics, and spatial proximity. We identified moderately strong correlations between teammate speeds (median Spearman ρ=0.78), with leader-follower reaction times typically very brief (median lag of 2 seconds). This implies highly coordinated team behavior rather than independent individual movements. Importantly, this coordination persisted when evaluated at decorrelated timescales, suggesting that synchrony reflects sustained behavioral coupling rather than short-timescale sampling artifacts. Additionally, teams often maintained close spatial proximity between searchers, with median separations of approximately 17 meters. Although there were occasionally large gaps, the general movement was cohesive and likely to maintain visual contact among teammates.

While our leadership analysis successfully elected leaders within each team, there were often ties in the cumulative-distance metric due to minimal temporal lag between searchers, suggesting that leaders may not always be positioned at the front. Leader-centrality calculations reveal that elected leaders often occupy central positions within the group. However, Team 4’s leader was centrally located only 14% of the time and exhibited minimal information transfer to followers, which can be seen in Figs 8 and 9. These observations could imply that leadership shifts or is context dependent within search teams. Importantly, we should emphasize that we see similar results for Team 4 even when transfer-entropy inputs differed from those used in leader election, underscoring the robustness of this finding.

### Searcher modeling implications

These results show some clear implications for modeling human searchers. Because of the symmetry in uphill and downhill speeds, we can use a single-magnitude exponential coefficient to represent slope penalties, simplifying their inclusion in models of mobility or cost-surfaces [26,27,62,63]. We should also use distinct baseline speeds (the flat-ground parameter *a*) between hasty and sweep searches, but maintain a common slope-sensitivity parameter (*b*) to avoid over-parameterization. Calculated coordination metrics, like cross-correlation magnitudes, reaction lags, and transfer entropy, could provide guidelines for relationship rules or probabilistic constraints in agent-based models, such as maximum separation thresholds, limited lag windows, or leader election using percent-ahead voting. Instead of assuming constant searcher speed in sweep-width and coverage calculators, we could use speeds adjusted by tactic and terrain (including the associated uncertainty) to thereby improve POD estimates. Finally, these empirically derived lags, pair-wise separations, and leadership patterns could serve as priors in Markov decision processes or other multi-agent decision frameworks used in SAR simulations [37,64–66].

Collectively, these adjustments pair naturally with recent advances in spacing/POD standardization (e.g., “head, belt, boots”) by providing the velocity side of the coverage equation [4,11,67]. Integrating both spacing and realistic speed distributions should reduce the ±15−25% POD variability observed when teams drift from planned parameters [67].

All incidents analyzed in this study occurred in Virginia and reflect terrain typical of the Mid-Atlantic region, including mixed forest, moderate elevation change, and established trail networks. While these environments include hilly and mountainous terrain, caution should be exercised when generalizing baseline speeds or slope coefficients to substantially different landscapes, such as arid desert or high-elevation western U.S. terrain.

That said, several of the patterns we observe are likely to extend beyond this specific setting. The relative difference in baseline speed between search tactics, the approximate symmetry in slope effects, and the strong coordination observed within teams all reflect general features of human movement and small-group dynamics rather than region-specific effects. As a result, we expect these relationships to remain useful when adapted to other SAR environments, even if the exact parameter values require recalibration.

Similar terrain-informed mobility modeling and GPS-based analyses are being developed in other SAR contexts, including mountainous regions in Europe, where both modeling and operational tools integrate terrain, movement, and search strategy considerations [68]. This suggests that empirically derived relationships of this type can transfer across settings when paired with locally relevant data. Future work should focus on validating and refining these parameters using datasets from a wider range of geographic and operational conditions.

## Conclusions

Using GPS tracks from 13 SAR incidents, we focused on two primary search tactics (hasty and sweep) and six small-group operations. In our statistical analysis, we: (1) performed bootstrapped segment-level Kolmogorov-Smirnov tests to compare speed distributions, (2) fit exponential speed-slope curves with two-level bootstrapping to obtain tactic-specific parameters, (3) used a nested ANOVA with random effects to test combined slope-tactic influences, and (4) quantified team coordination through Spearman correlations, time-lagged cross-correlation, and transfer entropy.

We found: (i) symmetric slope effects on speed, (ii) significantly higher baseline speeds for hasty searches, (iii) strong within-team coupling with short reaction lags, and (iv) directional information flow from elected leaders to followers. These empirically derived parameters and coordination metrics could provide actionable inputs for SAR movement models, cost-surface construction, and coverage/POD calculators. For a table of all summarized results, see S2 Table.

For future models of SAR searcher speeds, we suggest: (a) accounting for differences in baseline speed between hasty and sweep search strategies, (b) simplifying the effect of slope on speed with symmetrical exponential models, (c) identifying clear leadership roles using measured lags and separation constraints to improve coordinated search coverage, and (d) exploring other search tactics (e.g., tracking or expanding circle search) or team relationships (e.g., between humans and canines) to attain a more complete understanding of search and rescue dynamics. Integrating these behaviorally grounded parameters with standardized spacing and POD frameworks should improve both planning realism and operational outcomes.

## Supporting information

S1 Fig Team 2 search track coordinates. The search tracks for Team 2 shown over their common interpolated time from beginning (blue) to end (yellow), indicated by the right colorbar. The markers show the track starts (blue circles) and ends (red squares). The background shows the elevation of the region using the top colorbar to show the elevation range, where darker shades of gray represent higher elevations with respect to sea level.
(PDF)

S2 Fig Team 3 search track coordinates. The search tracks for Team 3 shown over their common interpolated time from beginning (blue) to end (yellow), indicated by the right colorbar. The markers show the track starts (blue circles) and ends (red squares). The background shows the elevation of the region using the top colorbar to show the elevation range, where darker shades of gray represent higher elevations with respect to sea level.
(PDF)

S3 Fig Team 4 search track coordinates. The search tracks for Team 4 shown over their common interpolated time from beginning (blue) to end (yellow), indicated by the right colorbar. The markers show the track starts (blue circles) and ends (red squares). The background shows the elevation of the region using the top colorbar to show the elevation range, where darker shades of gray represent higher elevations with respect to sea level.
(PDF)

S4 Fig Team 5 search track coordinates. The search tracks for Team 5 shown over their common interpolated time from beginning (blue) to end (yellow), indicated by the right colorbar. The markers show the track starts (blue circles) and ends (red squares). The background shows the elevation of the region using the top colorbar to show the elevation range, where darker shades of gray represent higher elevations with respect to sea level.
(PDF)

S5 Fig Team 6 search track coordinates. The search tracks for Team 6 shown over their common interpolated time from beginning (blue) to end (yellow), indicated by the right colorbar. The markers show the track starts (blue circles) and ends (red squares). The background shows the elevation of the region using the top colorbar to show the elevation range, where darker shades of gray represent higher elevations with respect to sea level.
(PDF)

S1 TableTeam search track metrics.Leader-follower pair metrics separated into 6 teams, where distance *d* is the separation between teammates in meters, speed ρ is the Spearman rank correlation between speeds, δ is the mean decorrelation time, lag is the reaction lag between teammates in seconds, |*r*| is the absolute speed cross-correlation magnitude, and centrality is the percentage of times the elected leader was near the center of each group. Note that *p* < 10^−3^ for all correlations. The mean decorrelation time, δ, for each team is the minimum value of *k* for *c*(*k*) < 1/*e*, and *c*(*k*) is the average autocorrelation function: $c(k)=\frac{1}{NT}\sum_{i=1}^{i=N}\sum_{t=1}^{T-k}(y_{i,t}-\bar{y_{i}})(y_{i,t+k}-\bar{y_{i}})$, where *N* is the total number of searchers in a team, *T* is the total time of the search, *y* is the speed time series, *i* is a team member, and *k* is the time lag.(PDF)

S2 Table Summary of key results across research questions (RQs). (PDF)
