# Supplementary material for: Speed, slope, and synchrony: Empirical insights into SAR searcher behavior
Source: PLoS One. 2026 Jun 15;21(6):e0339541. doi: 10.1371/journal.pone.0339541 (PMC13268166; doi:10.1371/journal.pone.0339541)
Supplement: S1 Fig — The search tracks for Team 2 shown over their common interpolated time from beginning (blue) to end (yellow), indicated by the right colorbar. The markers show the track starts (blue circles) and ends (red squares). The background shows the elevation of the region using the top colorbar to show the elevation range, where darker shades of gray represent higher elevations with respect to sea level. (PDF) [file pone.0339541.s001.pdf]

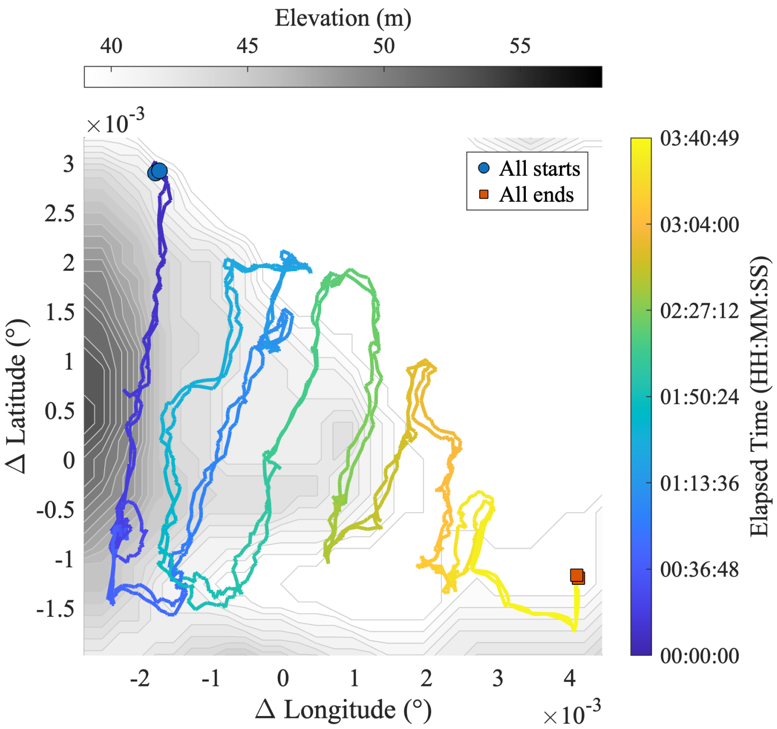

S1 Fig: The search tracks for Team 2 shown over their common interpolated time from beginning (blue) to end (yellow), indicated by the right colorbar. The markers show the track starts (blue circles) and ends (red squares). The background shows the elevation of the region using the top colorbar to show the elevation range, where darker shades of gray represent higher elevations with respect to sea level.
