# Supplementary material for: Speed, slope, and synchrony: Empirical insights into SAR searcher behavior
Source: PLoS One. 2026 Jun 15;21(6):e0339541. doi: 10.1371/journal.pone.0339541 (PMC13268166; doi:10.1371/journal.pone.0339541)
Supplement: S1 Table — Leader-follower pair metrics separated into 6 teams, where distance d is the separation between teammates in meters, speed ρ is the Spearman rank correlation between speeds, δ is the mean decorrelation time, lag is the reaction lag between teammates in seconds, |r| is the absolute speed cross-correlation magnitude, and centrality is the percentage of times the elected leader was near the center of each group. Note that p < 10−3 for all correlations. The mean decorrelation time, δ, for each team is the minimum value of k for c(k) < 1/e, and c(k) is the average autocorrelation function: c(k)=1NT∑i=1i=N∑t=1T−k(yi,t−yi¯)(yi,t+k−yi¯), where N is the total number of searchers in a team, T is the total time of the search, y is the speed time series, i is a team member, and k is the time lag. (PDF) [file pone.0339541.s006.pdf]

S1 Table: Leader-follower pair metrics separated into 6 teams, where distance d is the separation between teammates in meters, speed ρ is the Spearman rank correlation between speeds, δ is the mean decorrelation time, lag is the reaction lag between teammates in seconds, $|r|$ is the absolute speed cross-correlation magnitude, and centrality is the percentage of times the elected leader was near the center of each group. Note that $p < 10^{-3}$ for all correlations. The mean decorrelation time, δ, for each team is the minimum value of k for $c(k) < 1/e$, and $c(k)$ is the average autocorrelation function: $c(k) = \frac{1}{NT}\sum_{i=1}^{i=N}\sum_{t=1}^{T-k}(y_{i,t} - \bar{y_i})(y_{i,t+k} - \bar{y_i})$, where N is the total number of searchers in a team, T is the total time of the search, y is the speed time series, i is a team member, and k is the time lag.

**Leader**	**Follower**	**Mean d**	**Max d**	**Speed ρ**	**Decorr. δ**	**Lag**	**Speed $\lvert r\rvert$**	**Centrality**
Leader 1	Follower A	10.7 m	42.8 m	0.85	44 s	2 s	0.63	87.4%
	Follower B	16.3 m	47.4 m	0.87		5 s	0.77	
Leader 2	Follower A	9.6 m	42.2 m	0.74	36 s	0 s	0.60	41.4%
Leader 3	Follower A	15.9 m	40.8 m	0.75	36 s	-4 s	0.58	57.3%
Leader 4	Follower A	27.1 m	99.9 m	0.49	132 s	7 s	0.54	14.6%
	Follower B	14.3 m	88.7 m	0.66		-2 s	0.64	
	Follower C	32.3 m	119.0 m	0.53		-10 s	0.55	
Leader 5	Follower A	15.1 m	54.1 m	0.78	87 s	-2 s	0.51	71.9%
	Follower B	14.9 m	62.3 m	0.74		22 s	0.44	
Leader 6	Follower A	26.3 m	211.8 m	0.86	150 s	7 s	0.71	65.9%
	Follower B	32.0 m	207.3 m	0.84		-1 s	0.74	
	Follower C	25.3 m	213.0 m	0.88		1 s	0.74	
