# Supplementary material for: Speed, slope, and synchrony: Empirical insights into SAR searcher behavior
Source: PLoS One. 2026 Jun 15;21(6):e0339541. doi: 10.1371/journal.pone.0339541 (PMC13268166; doi:10.1371/journal.pone.0339541)
Supplement: S2 Table — (PDF) [file pone.0339541.s007.pdf]

S2 Table: Summary of key results across research questions (RQs).

Research Question	Metric	Result	Practical Takeaway
Slope effect (RQ 1)	Median speed	Uphill: 0.48 m/s (IQR 0.26 − 0.77)<br>Downhill: 0.52 m/s (IQR 0.30 − 0.85)<br>KS $p = 0.093$	No significant difference; slope effects are symmetric.
	Exponential fit, $v = ae^{bx}$	$a_{\text{up}} = 1.05$ [95% CI 0.90 − 1.19]<br>$a_{\text{down}} = 1.13$ [95% CI 0.94 − 1.30]<br>$b_{\text{up,down}} \approx \pm 3.5$	A single slope coefficient magnitude ($\|b\| \approx 3.5$) can represent both uphill and downhill.
Search type (RQ 2)	Median speed	Hasty: 0.53 m/s (IQR 0.26 − 0.87)<br>Sweep: 0.39 m/s (IQR 0.20 − 0.63)<br>KS $p < 10^{-3}$	Hasty searches are faster; sweep searches are slower but steadier.
	Exponential fit, $v = ae^{bx}$	Hasty: $a_{\text{up}} = 1.11$, $a_{\text{down}} = 1.17$<br>Hasty: $b_{\text{up}} = -3.21$, $b_{\text{down}} = 2.94$<br>Sweep: $a_{\text{up}} = 0.93$, $a_{\text{down}} = 0.97$<br>Sweep: $b_{\text{up}} = -3.57$, $b_{\text{down}} = 3.48$	Search type mainly affects baseline speed (a), not slope sensitivity (b); no significant difference in the effect of slope on speed in both hasty and sweep types.
RQ 2 + slope interaction	Nested ANOVA	Type effect: $F(1, 41) = 4.85$, $p = 0.03$<br>Slope effect: $F(2, 82) = 15.51$, $p < 10^{-3}$<br>No interaction ($p = 0.38$)	Hasty speed > Sweep speed across all slopes; slope affects speed with downhill fastest; slope effects consistent across tactics.
Team coordination (RQ 3)	Speed correlation	Median $\rho = 0.78$ (IQR 0.71 − 0.85)	Teammates' speeds are moderately coupled.
	Reaction lag	Median 2 s (IQR −2 − 6); ∼90% < 10 s	Teams react to each other almost instantly.
	Cross-correlation	$\|r\| = 0.64$ (IQR 0.55 − 0.71)	Teams show strong speed coupling.
	Separation distance	Median 17 m (IQR 15 − 26)	Teams maintain close visual contact.
	Leadership	Leaders in all 6 teams; TE positive in 4/6	Clear leader-follower information flow; some teams show shared leadership.
